# Comparison of glucosamine sulfate and a polyherbal supplement for the relief of osteoarthritis of the knee: a randomized controlled trial [ISRCTN25438351]

**DOI:** 10.1186/1472-6882-7-34

**Published:** 2007-10-31

**Authors:** Komal Mehta, Jayesh Gala, Surendra Bhasale, Sattayasheel Naik, Millind Modak, Harshad Thakur, Nivedita Deo, Mark JS Miller

**Affiliations:** 1 Vedic Lifesciences, Pvt. Ltd., Mumbai, India; 2 A-1, Om Kamal Bldg, Mumbai, India; 3 Diamond Hospital, Mumbai, India; 4 Naik Hospital, Pune, India; 5 Yogesh Hospital, Pune, India; 6 Dept. of Health Services Studies, Tata Institute of Social Sciences, Mumbai, India; 7 Albany Medical College, Albany, USA

## Abstract

**Background:**

The efficacy and safety of a dietary supplement derived from South American botanicals was compared to glucosamine sulfate in osteoarthritis subjects in a Mumbai-based multi-center, randomized, double-blind study.

**Methods:**

Subjects (n = 95) were screened and randomized to receive glucosamine sulfate (n = 47, 1500 mg/day) or reparagen (n = 48, 1800 mg/day), a polyherbal consisting of 300 mg of vincaria (*Uncaria guianensis*) and 1500 mg of RNI 249 (*Lepidium meyenii*) administered orally, twice daily. Primary efficacy variable was response rate based on a 20% improvement in WOMAC pain scores. Additional outcomes were WOMAC scores for pain, stiffness and function, visual analog score (VAS) for pain, with assessments at 1, 2, 4, 6 and 8 weeks. Tolerability, investigator and subject global assessments and rescue medication consumption (paracetamol) were measured together with safety assessments including vital signs and laboratory based assays.

**Results:**

Subject randomization was effective: age, gender and disease status distribution was similar in both groups. The response rates (20% reduction in WOMAC pain) were substantial for both glucosamine (89%) and reparagen (94%) and supported by investigator and subject assessments. Using related criteria response rates to reparagen were favorable when compared to glucosamine. Compared to baseline both treatments showed significant benefits in WOMAC and VAS outcomes within one week (P < 0.05), with a similar, progressive improvement over the course of the 8 week treatment protocol (45–62% reduction in WOMAC or VAS scores). Tolerability was excellent, no serious adverse events were noted and safety parameters were unchanged. Rescue medication use was significantly lower in the reparagen group (p < 0.01) at each assessment period. Serum IGF-1 levels were unaltered by treatments.

**Conclusion:**

Both reparagen and glucosamine sulfate produced substantial improvements in pain, stiffness and function in subjects with osteoarthritis. Response rates were high and the safety profile was excellent, with significantly less rescue medication use with reparagen. Reparagen represents a new natural productive alternative in the management of joint health.

**Trial registration:**

Current Controlled Trials ISRCTN25438351.

## Background

Osteoarthritis is a debilitating condition that is of growing concern and significance given shifts in population profiles towards the aged in most developed countries [1,2]. In addition to demographic considerations, osteoarthritis remains a therapeutic challenge and it is not uncommon for patients to complement a pharmaceutical approach with nutraceuticals, herbals, acupuncture and other complementary medicine modalities [3-5]. While the current pharmaceutical options, usually non-steroidal anti-inflammatory drugs (NSAIDs), primarily focus on symptom relief [6,7], some complementary medicines have the potential to alter the disease process [5,8,9]. The current goals are to limit or retard joint destruction, however the ultimate therapeutic endpoint is to achieve restoration of joint form and function.

Glucosamine, alone or with the more complex form of matrix, chondroitin, is the most commonly used nutraceutical option, and has been subject to significant scrutiny [10]. Two recent large studies, the Glucosamine/Chondroitin Arthritis Intervention Trial (GAIT) and the Glucosamine Unum In Die (once a day) Efficacy (GUIDE) have yielded conflicting results [11,12]. The GAIT study used glucosamine hydrochloride administered three times a day and did not yield positive endpoints when compared to placebo [9]. Glucosamine hydrochloride is less commonly used than glucosamine sulfate and lacks supportive evidence for efficacy [13,14]. On the other hand, the once-a-day regimen of glucosamine sulfate in the GUIDE study, demonstrated significant improvements over placebo control [12]. The glucosamine sulfate formulation is more popular and is approved as a prescription formulation in Europe. Additionally, several long-term studies indicate that it may potentially delay, unlike NSAIDs, structural changes to the joint in osteoarthritis [8,9]. Nevertheless, efficacy and response rates to glucosamine and other treatment modalities are variable [14,15] and we remain distant from the goal of defining safe interventions that improve joint architecture. Glucosamine while regarded as safe does have potential complications for some individuals that limit its use [15,16]. As the majority of glucosamine is produced from seafood sources there is a concern for potential allergic responses. Nevetheless glucosamine is generally considered safe although there is debate as to whether glucosamine may promote insulin resistance, raise glucose levels or vascular perturbations that may accelerate atherosclerosis [16-19]. This has contributed to the search for additional options.

To this end we have been evaluating botanicals that may offer a disease modifying approach based on their redox related actions on gene expression. Reparagen^®^, is a polyherbal based on a blend of extracts from an Amazonian tea (*Uncaria guianensis*) and an Andean vegetable (*Lepidium meyenii*), that has a unique profile and mechanism of action [20]. Extracts of *Uncaria guianensis *have been shown to suppress NF-κB, a transcription factor that regulates a multitude of cytokines, chemokines, and enzymes that contribute to the inflammatory process [21-24]. Traditionally used in the Amazon and South America for arthritis and other forms of chronic inflammation, we have demonstrated that low doses (100 mg/day) of vincaria offer rapid symptomatic relief of osteoarthritis [25]. The present trial is an extension of that observation using a three fold higher dose and an additional value added ingredient RNI 249 that promotes cartilage production of IGF-1 [20]. These actions of RNI 249, observed in human cartilage explants, were enhanced by co-administration of vincaria [20]. This preclinical research and development was funded by the National Institutes of Health (USA, R43 AG024733-01), and resulted in the investigational product used in this trial.

IGF-1 is an anabolic growth factor that contributes to cartilage repair and growth [26-30]. Inflammation suppresses the local expression of IGF-1 in cartilage [20,26,27,29], as in other tissues [31-34], resulting in a catabolic state and impaired tissue repair [31-36]. Thus, therapies that either directly replenish cartilage IGF-1 levels or enhance local IGF-1 production offer a potential new approach to improving joint health. Glucosamine has been postulated to limit cartilage degradation at high concentrations (mM) in cartilage explants [37,38], but in contrast to reparagen, glucosamine also appears to reduce anabolic processes together with catabolic pathways, suggesting that its potential for cartilage matrix repair may be limited [37].

This preliminary clinical investigation was not designed to evaluate improvements joint architecture; it was too brief for a meaningful evaluation. However, the focus was to determine in a controlled clinical investigation if reparagen offered relief of osteoarthritis symptoms, using glucosamine sulfate as the comparator. The protocol was for two months, but was highly detailed within that design with assessments at weeks 1, 2, 4, 6 and 8 in addition to the entry and baseline measurements. Safety metrics were also assessed at these time-points (vital signs) and laboratory tests at the beginning and conclusion of the study.

## Methods

### Research Design

This randomized, double-blind, positive control, multi-center trial was performed in Mumbai, India with approval by the Institutional Ethics Committee of the K.J. Somaiya Medical College & Hospital, and was in compliance with the Helsinki Declaration.

### Participants

Subjects were recruited from six centers in Mumbai, India. These centers represent a combination of hospitals and clinics specializing in orthopedics. Inclusion criteria were ambulatory, adult patients of either sex and greater than 20 years of age with mild to moderate osteoarthritis as determined by radiological examination and ARA functional class II or III, and Kellgren Lawrence classification grade II or grade III, and a baseline functional assessment of overall pain of at ≥40 mm and ≤80 mm, on a 100 mm Visual Analog Scale (VAS).

Exclusion criteria were: existence of other forms of arthritis, arthroscopy of either knee within the past year, administration of intra-articular steroids with the past 3 months or hyaluronic acid in the last 9 months, pregnancy or lactating women or women not taking adequate birth control measures, presence of any concomitant unstable disease or abnormality of any clinically relevant laboratory test, evidence of severe renal or hematologic disease, cardiac insufficiency, moderate to severe neuropathy, and unwillingness to come to regular follow-up visits for the length of the study.

A Fixed Allocation Randomization procedure using an algorithm was used to assign interventions to the participants with a pre-specified probability and on a per project basis after subjects passed the screening procedures.

### Treatments

The duration of treatment was 8 weeks, with either glucosamine sulfate (1500 mg) or reparagen (1800 mg) both administered as two capsules, twice a day orally before meals. Glucosamine sulfate 2 KCl (99.1% purity) was supplied by a local manufacturer (Healers Nutraceuticals, Pvt. Ltd., Chennai, India) and reparagen was supplied by Rainforest Nutritionals, Inc. (Raleigh, NC, USA). The twice a day dosing regimen was chosen to facilitate compliance within the target population. Previously glucosamine sulfate has been shown to be effective when administered using either at once and three times a day dosing regimens [8,9,12,39-42] suggesting that the timing of administration is not a constraint for efficacy. Ethnomedical evidence related to the constituents within reparagen favor twice a day administration, and given these issues, twice a day dosing was chosen to be optimal. The rescue medication was paracetamol (acetominophen) provided as 500 mg tablet, with daily dosing not to exceed 3 tablets a day (1500 mg) for the first 4 weeks and 2 tablets (1000 mg) for the last 4 weeks. A framework for the present trial was a previous trial in osteoarthritis with the vincaria component alone [25], administered at a three-fold lower dose (100 mg). This former study demonstrated significant improvements in pain within one week, with progressive improvements over the four week treatment protocol [25].

The test agent Reparagen^® ^is a proprietary blend of two natural products – vincaria (300 mg) and RNI 249 (1500 mg). Vincaria is an extract of *Uncaria guianensis *that is traditionally used to treat chronic inflammation including arthritis [43]. RNI 249 is an extract of *Lepidium meyenii*, an Andean vegetable and food staple in the Cruciferous family. This vegetable has been harvested for nearly 6000 years to manage the health compromising effects of high altitude [44,45].

Uniformity was maintained in both treatment groups in terms of capsule weight, size, color, bottle filling, labeling, and packaging. Treatments were packaged in red gelatin capsules and packaged in wide mouthed white, opaque bottles with screw caps in a clean room. Investigators were provided with blinding chits containing patient codes along with their treatment group (alphabetical). In case of a serious adverse event investigators were instructed to inform the monitors and then only unblind the treatment group of the subject if the event appeared to be agent related in order to address the necessary treatment. Compliance was manually checked by study monitors by way of pill count (study and rescue medication)

### Primary Efficacy Variable – Response to Treatment

We used a modified version of the WOMAC (Western Ontario and McMaster) Universities Osteoarthritis Index as a disease-specific measure of health status. The changes reflect the specific needs and cultural considerations of this population and have been detailed before [46]. There are three sections that deal with pain (5 questions), stiffness (2 questions), and function or performance (21 questions). Each question had a response on a scale of 0 – 4, with 0 representing none, 1 slight, 2 moderate, 3 severe, 4 extreme.

As reported in the GAIT clinical trial investigating glucosamine hydrochloride and chondroitin sulfate in osteoarthritis subjects [11], we used response rate to treatment as a primary efficacy variable based on the WOMAC A or pain criteria. For a subject to be deemed a responder there must be a 20% reduction in their basal WOMAC pain score [11]. In addition, given that this criteria is not stringent we sought additional metrics based on alleviation of WOMAC or VAS assessments of pain to determine if a subject responded to therapy. These included (1) a 50% reduction of WOMAC pain score (2) a combination of a 20 mm reduction in VAS pain and a 50% reduction in WOMAC pain assessments (3) a 10 mm reduction in VAS pain measurements and a 20% reduction in WOMAC pain assessments. These additional metrics are similar to the OMERACT-OARSI criteria with the exception that these normally include a VAS for function as well as pain. However, in this study we only used a VAS approach for pain and not function and so we limited the possible responder entry criteria to pain metrics.

### Secondary Efficacy Variables

A VAS (visual analog score) assessment of pain was included as a secondary efficacy variable. With this assessment a line of 100 mm is drawn to measure the individuals pain status, with 0 representing no pain, and 100 being unbearable pain. Patients marked on this line the relevant amount of pain they were experiencing and the value was noted by the investigator, in mm.

The individual components of the WOMAC scale – pain, function, performance and total were used as secondary outcome assessments. Treatment effectiveness was assessed by both investigators and subjects using a scale that incorporated the following elements: Excellent – complete relief of symptoms; Good – partial relief of symptoms; Fair – minimal relief of symptoms; Poor – no relief of symptoms; Very Poor – worsening of symptoms.

Tolerability was assessed in three categories. Good – no side effects; Fair – mild to moderate side-effects; Poor – severe side-effects and withdrawal of therapy. Measurements of these secondary efficacy variables were made at the end of the treatment protocol.

### Rescue Medicine Consumption

Paracetamol was used as the rescue medication and dispensed as 500 mg tablets with a maximum of 3 tablets allotted per day for the first 4 weeks, and 2 tablets per day for the last 4 weeks. Subjects were required to return the paracetamol containers at each follow-up visit for counting and renewal for the next phase of the study. Instructions were to use paracetamol strictly for rescue purposes. This dose of paracetamol (1500 to 1000 mg) per day was substantially less than the 4000 mg maximum allowed in the GAIT study [11] and the 3000 mg used as an investigational arm of the GUIDE study [12]. This was approved by the institutional review board. The advantage of this approach is that it helped to diminish the complication that rescue medication may exert on the observations.

### Serum IGF-1

While not a primary or secondary efficacy variable, serum IGF-1 levels were measured in each subject before and at the conclusion of the trial. The rationale for this measurement was based on our preliminary ex vivo work with human cartilage, inflammatory cytokines and reparagen [20]. Blood was drawn between 8 and 10 in the morning, and subjects were not fasted.

### Data Quality Assurance

All investigators were informed of ICH-GCP guidelines. Data quality, study execution monitoring was performed by individuals independent of subject contact and treatment assessment.

### Statistical Analysis

Data was analyzed by a statistician who was blind to treatments using the following tests – Chi squared test, ANOVA, paired and unpaired t tests, Bonferroni, Dunnett's and Tukey's test as appropriate. SPSS 11.5, PEPI, EPI INFO 2000 and MS Excel. Statistical significance was taken at the 95% confidence level. Results are expressed as the mean ± SEM. All efficacy analyses were performed on an intention-to-treat basis. Safety metrics were assessed on a paired basis (baseline and completion of the protocol).

## Results

### Patient Randomization and Disposition

Descriptions of the entry profile for subjects in each treatment group are contained within Table 1, and demonstrate an effective randomization process. Reflecting the general demographic profile of the osteoarthritis, the majority of the subjects were female: glucosamine (75%), reparagen (75%). Age was also comparable in both treatment groups (glucosamine 55.1 ± 1.6 years, reparagen 51.9 ± 1.8 years).

**Table 1:** Baseline characteristics of treatment groups

	**Glucosamine**	**Reparagen**
**N Started (completed)**	47 (41)	48 (38)
**Age (years)**	55.1 ± 1.6	51.9 ± 1.8
**Gender (F:M %)**	75 : 25	75 : 25
		
**ARA Functional Class II:III, %**	79 : 21	71 : 29
**Kellgren Lawrence Criteria Grade 2:3, %**	79 : 21	75 : 25
		
***Screening***		
**WOMAC Pain**	8.1 ± 0.4	8.9 ± 0.5
**WOMAC Stiffness**	3.8 ± 0.2	4.0 ± 0.2
**WOMAC Function**	35.4 ± 2.0	37.2 ± 1.8
**WOMAC Total**	47.1 ± 2.5	50.1 ± 2.3
		
**VAS Pain**	65.4 ± 1.7	65.6 ± 1.5

Data expressed as a % of total or mean ± SEM. None of the entry assessments were significantly different between the glucosamine sulfate or reparagen groups.

Disease status upon entry was also effectively randomized between groups (Table 1) using both ARA Functional Class and Kellgren Lawrence Criteria grade as the means for quantifying disease severity. Both measurements revealed that the majority of subjects had grade II disease, and this was similar in both treatment groups. Using Chi square analysis no statistical difference in the entry disease status was evident amongst the treatment groups using either Kellgren Lawrence or ARA Functional Criteria.

**Figure 1:**
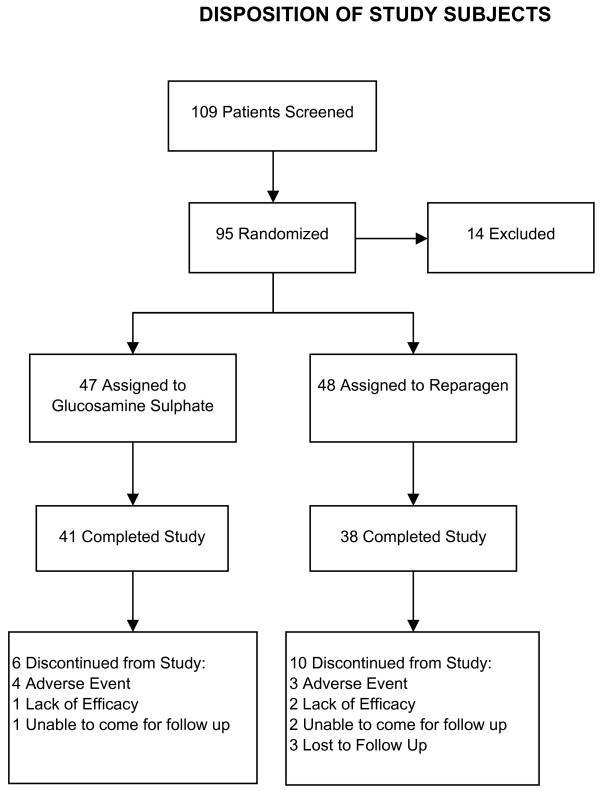
Study profile including enrollments and outcomes.

Drop-outs from the study were similar in both groups, disposition of subjects are described in Figure 1. There were some drop-outs due to lack of efficacy and pain associated with the condition between baseline measurements and the first two weeks of treatment. Subsequent losses were largely due to a failure to come for follow-up or for unrelated conditions and adverse events necessitating withdrawal. These adverse events were deemed to be unrelated to the treatments and included single cases of accident, loose bowel motions, hepatitis, gastric ulcer and viral fever. In the glucosamine group a serious adverse event at week 6, specifically gallstones requiring surgery and this was deemed to be unrelated to the treatment. In the glucosamine group 87% of the recruited subjects successfully completed the study, and for reparagen 79% completed the study.

### Safety Variables – Laboratory

All laboratory tests were unchanged from baseline values to week 8 for both treatment groups with the following exception: there was a slight reduction in ESR at week 8 in the reparagen treated group (p = 0.02), however the values remained within normal limits for this population and the result was viewed as not being clinically relevant. Details of all assessments are described in Table 2.

**Table 2:** Laboratory-based evaluations of safety

**Test**	**Glucosamine **Wk. 0	**Glucosamine **Wk. 8	**Reparagen **Wk. 0	**Reparagen **Wk. 8
**Neutrophils %**	63.46 ± 1.25	63.54 ± 1.26	62.19 ± 1.32	62.38 ± 1.27
**Lymphocytes %**	31.73 ± 1.21	32.00 ± 1.27	32.27 ± 1.18	32.27 ± 1.21
**Monocytes %**	1.17 ± 0.14	1.32 ± 0.15	1.11 ± 0.13	1.16 ± 0.15
**Eosinophils %**	3.54 ± 0.36	3.22 ± 0.30	4.11 ± 0.47	3.19 ± 0.41
**Basophils %**	0.02 ± 0.02	0 ± 0	0 ± 0	0 ± 0
**White blood cells mm**^3^	7929 ± 268	7515 ± 222	7753 ± 303	7496 ± 262
**Red Blood Cells mm**^3^	4.06 ± 0.10	4.04 ± 0.08	4.13 ± 0.11	4.15 ± 0.08
**Hemoglobin gm/dl**	12.02 ± 0.3	12.00 ± 0.2	11.84 ± 0.3	11.92 ± 0.2
**Erythrocyte Sedimentation Rate mm**	33.0 ± 3.6	30.3 ± 3.0	33.9 ± 3.4	28.5 ± 3.1a
**SGPT IU/L**	26.3 ± 2.6	23.6 ± 1.7	22.9 ± 2.1	19.9 ± 1.3
**Creatinine mg/dl**	0.97 ± 0.03	0.93 ± 0.03	1.17 ± 0.21	0.93 ± 0.03

Data expressed as a mean ± SEM.

There was no difference between baseline and week 8 values for the treatment groups with the exception of erythrocyte sedimentation rate as denoted by the letter a (p < 0.05). As the analysis was done on a paired basis completion of the protocol was necessary, hence the analysis is per protocol, glucosamine n = 41, reparagen n = 38.

### Safety Variables – Vital Signs

Blood pressure, respiration rate and pulse rate were measured at screening, baseline, and at weeks 1, 2, 4, 6 and 8 of treatment. Results are depicted in Table 3. Using ANOVA there was no significant alterations of these values with treatment.

**Table 3:** Vital Signs

**Vital Signzz**	**Glucosamine Wk. 0**	**Glucosamine Wk. 8**	**Reparagen Wk. 0**	**Reparagen Wk. 8**
**Pulse Rate**	76.0 ± 1.0	76.8 ± 0.7	78.1 ± 1.2	75.4 ± 1.0
**Systolic BP**	130 ± 3	128 ± 2	128 ± 2	127 ± 1
**Diastolic BP**	81 ± 1	80 ± 1	83 ± 1	81 ± 1
**Respiration Rate**	17.8 ± 0.4	17.7 ± 0.4	17.3 ± 0.3	17.9 ± 0.4

Data expressed as a mean ± SEM. Units of measure were: Pulse rate (beats per min), systolic and diastolic blood pressure (BP, mm Hg), respiration rate (breaths per minute). There were no significant differences between baseline and week 8 values with these treatments. As the analysis is on a paired basis before and after completing the protocol the analysis is per protocol, glucosamine n = 41, reparagen n = 38.

### Primary Efficacy Variable – Response to Treatment

**Figure 2:**
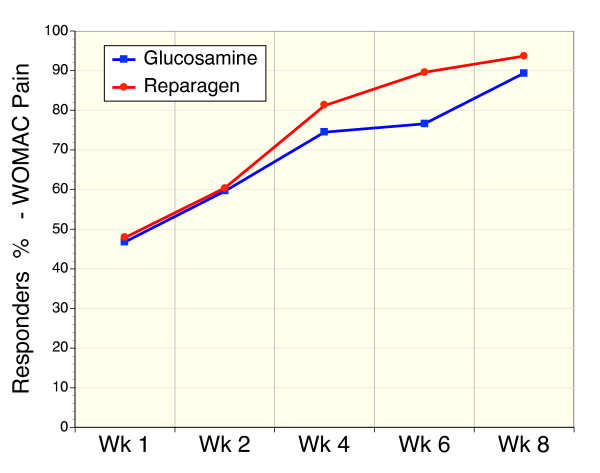
Sequential response rates as determined by a 20% reduction in the WOMAC pain scores in reparagen (red, n = 48) and glucosamine sulfate (blue, n = 47).

Response rate was calculated in accordance with the method described in the GAIT study [11]. This method uses a 20% reduction in WOMAC pain as the primary assessment of a response to treatment. Using this criteria both treatments were associated with a large proportion of responders within one week of treatment (reparagen 47.9%; glucosamine 46.8%). Response to both treatments continued to increase for the duration of the study protocol (Fig. 2). At week 4 the response rates were similar with both treatments: reparagen 81.2%, glucosamine 74.5% and at the conclusion of the study at week 8: reparagen 93.7%, glucosamine 89.4% (Table 4).

**Table 4:** Response rates of subjects to treatment based on pain assessments.

	**4 Weeks**		**8 Weeks**	
*Criteria*	*Glucosamine* *(n = 47)*	*Reparagen* *(n = 48)*	*Glucosamine* *(n = 47)*	*Reparagen* *(n = 48)*

20% decrease in WOMAC pain	74.5	81.2	89.4	93.7
20% decrease in WOMAC pain & 10 mm VAS Pain	66.0	68.7	85.1	91.7
50% decrease in WOMAC pain	38.2	58.3*	72.3	77.1
50% decrease in WOMAC pain & 20 mm VAS Pain	25.5	37.5	63.8	72.9

Percentage of subjects meeting various criteria for response to treatment. Results between reparagen (n = 48) and glucosamine sulfate (n = 47) were not significantly different using these criteria except where denoted by the * (P = 0.05).

We also used a more rigorous assessment of response rates based on WOMAC pain scales than that used in the GAIT study [11], specifically a 50% reduction in WOMAC pain as the definition of a response to treatment as opposed to a 20% reduction (Table 4). Using this more stringent assessment the response rate to reparagen was significantly greater than glucosamine at week 4 (p = 0.05) but not at week 8.

We also sought to determine response rates to treatment using the response criteria similar to that outlined by OMERACT-OARSI [11,12]. However, as VAS measures were for pain and not pain and function, these assessments do not follow the precise OMERACT-OARSI criteria. Nevertheless they provide a useful tool for assessing response rates, in addition to the approaches described above. Two levels of response were calculated based on a combination of WOMAC and VAS metrics of pain and results are summarized in Table 4. Firstly the more rigorous criteria for response to treatment was determined by a subject experiencing a 50% improvement in WOMAC pain subscale and a reduction of VAS pain by 20 mm. Using this criteria response rates tended to be greater for reparagen but differences were not significant (Table 4). The second level of determining response rates was less rigorous, similar to the GAIT criteria, and used a 20% reduction in WOMAC pain subscale and a 10 mm reduction in VAS pain. Similar trends to the other criteria were noted (Table 4). These metrics of response to therapy indicate that both reparagen and glucosamine sulfate produced time-dependent response rates based on improvements in pain assessments (WOMAC and VAS).

### Secondary Efficacy Variable – WOMAC

**Figure 3:**
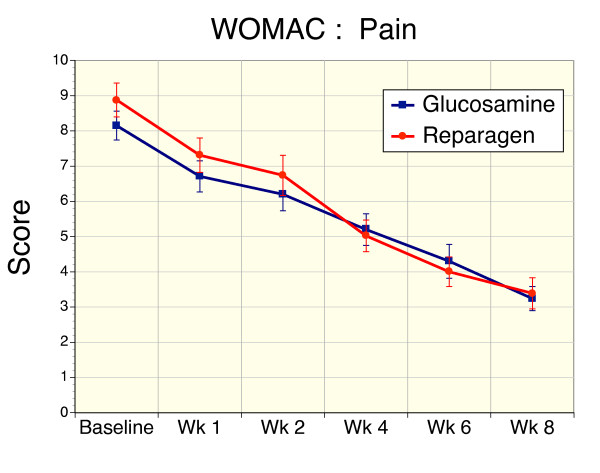
Sequential changes in WOMAC pain scores for reparagen (red, n = 48) and glucosamine sulfate (blue, n = 47). Both treatments resulted in a significant reduction in WOMAC pain levels within one week of treatment (p < 0.001) compared to baseline values. Sustained administration resulted in a time-dependent decrease in pain scores (p < 0.001)

**Figure 4:**
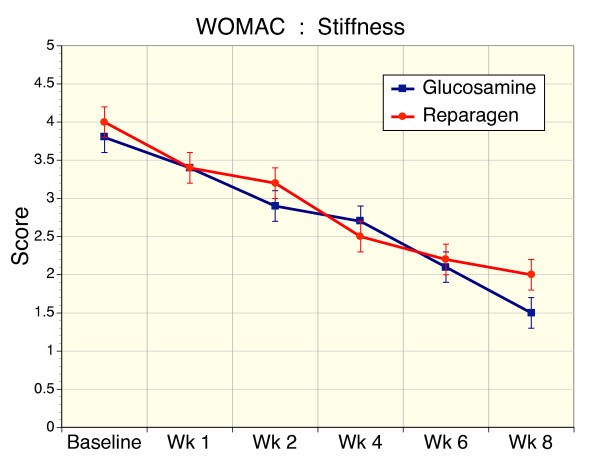
Sequential changes in WOMAC stiffness scores for reparagen (red, n = 48) and glucosamine sulfate (blue, n = 47). Both treatments resulted in a significant reduction in baseline stiffness scores from week 2 onwards (p < 0.001), but only the reparagen treated group was significant at week 1 (p < 0.01). Sustained administration resulted in a time-dependent decrease in stiffness scores (p < 0.001)

**Figure 5:**
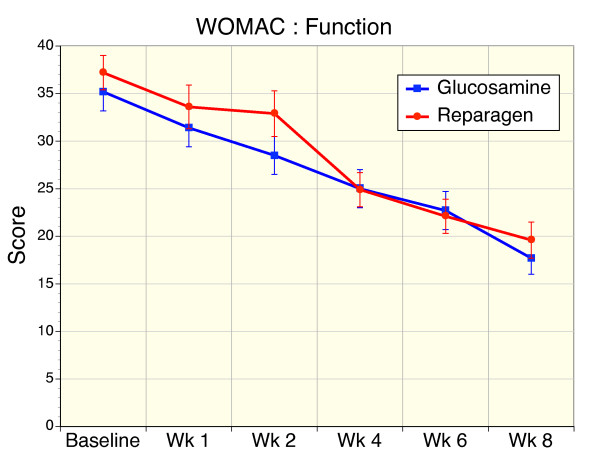
Sequential changes in WOMAC function scores for reparagen (red, n = 48) and glucosamine sulfate (blue, n = 47). Both treatments resulted in a significant improvement in function within one week (reparagen p < 0.01, glucosamine p < 0.05) with continued improvements with sustained administration (p < 0.001).

**Figure 6:**
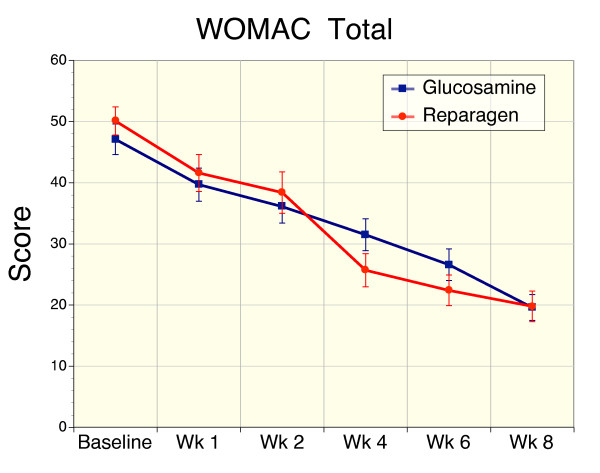
Sequential changes in total WOMAC scores (pain, stiffness and function) for reparagen (red, n = 48) and glucosamine (blue, n = 47). Both treatments resulted in a significant reduction in baseline WOMAC total scores from week 1 (reparagen p < 0.01, glucosamine p < 0.05), with further improvements with sustained administration (p < 0.001).

Baseline disease activity as defined by WOMAC pain (Fig 3), stiffness (Fig 4) and function or performance (Fig 5), or total WOMAC scores (Fig. 6) were comparable and not significantly different in the glucosamine or reparagen treatment groups. With both treatments these assessments were significantly improved within one week of treatment (p < 0.05) for the individual components of WOMAC – pain, stiffness, function (Figs. 3, 4, 5) or the total WOMAC assessment (Fig. 6) with the following exceptions. The glucosamine sulfate group did not achieve a significant reduction in WOMAC stiffness scores until week 2. It is also clear that with continued administration of the test agents there were steady improvements in these assessments of disease activity (p < 0.001, repeated measures ANOVA). However, there were no significant differences in the magnitude of these changes between the two treatment groups; both treatments producing comparable benefits over the course of this investigation.

For WOMAC pain scores the overall benefit was a 60% reduction for glucosamine and 62% for reparagen. For WOMAC stiffness scores were reduced with glucosamine by 61% reduction and reparagen 51% at the end of 8 weeks of treatment. For WOMAC functional assessments, glucosamine elicited a 62% improvement from baseline and reparagen 61% at 8 weeks. Similarly, responses to treatment as defined by percentage reduction in Total WOMAC scores were similar for glucosamine (58%) and reparagen (60%).

### Secondary Efficacy Variable – VAS Pain

**Figure 7:**
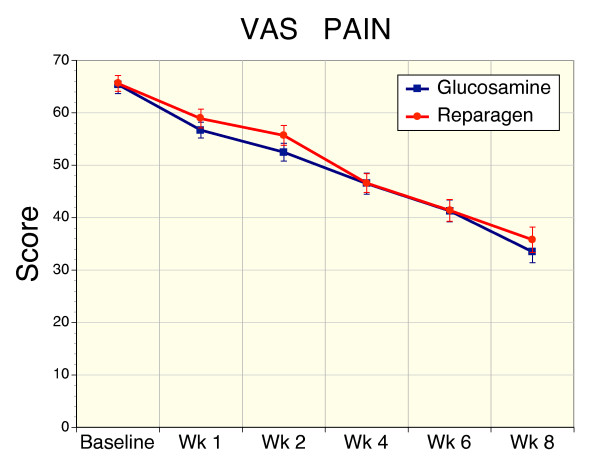
Sequential changes in VAS pain scores for reparagen (red, n = 38) and glucosamine sulfate (blue, n = 41). Both treatments resulted in a significant reduction in baseline VAS pain scores within one week (p < 0.01), with further reductions with sustained administration (p < 0.001).

Pain status in the glucosamine and reparagen groups as determined by VAS, were comparable at recruitment (Table 1) and baseline (Fig. 7). Treatment resulted in a reduction in VAS pain scores, with significance noted at week 1 in both treatment groups (p < 0.05), and a steady further decline in VAS pain values over the 8 week course of treatment (Fig. 7). At the conclusion of the study there was a 49% reduction in VAS pain in the glucosamine group and a 45% reduction in the reparagen group. There was no significant difference between the reparagen and glucosamine groups in the magnitude of changes in the VAS assessment of pain.

### Secondary Efficacy Variables – Global Assessments, Tolerance

Global assessments of the treatment were obtained at the conclusion of the 8 week treatment protocol, using both investigator and subject perspectives. Investigators reported a score of excellent (the highest level) for 97% of the glucosamine treated subjects, and 92% for reparagen. These values were not significantly different. Subjects' assessments were based on a willingness to continue with the treatments. For both reparagen and glucosamine sulfate 95% of subjects stated they would like to continue treatment. Tolerability was rated by subjects as Good in 100% of the reparagen treated group, and 98% in the glucosamine sulfate group.

### Serum IGF-1

Serum IGF-1 levels were assessed at baseline and at the conclusion of the 8 weeks study. Baseline values for both glucosamine (93.4 ± 33.4 ng/ml) and reparagen (89.1 ± 32.6 ng/ml) treatment groups were comparable, indicating effective randomization. With both treatments there was a 5% increase in serum IGF-1 (glucosamine 98.2 ± 36.6, reparagen 93.2 ± 38.2 ng/ml), which was not statistically significant.

### Use of Rescue Medication

**Figure 8:**
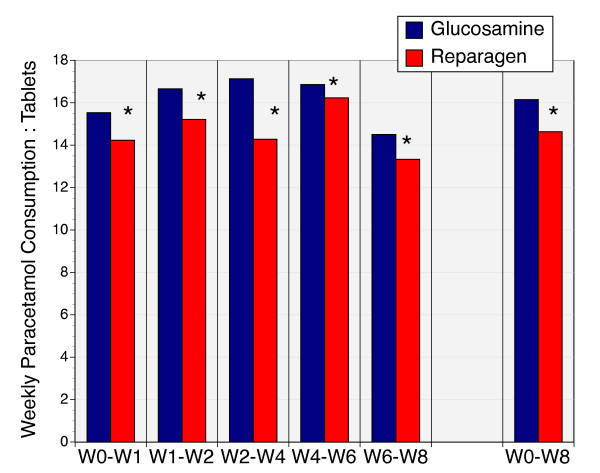
The average weekly consumption of paracetamol (acetominophen) in reparagen (red, n = 38) and glucosamine sulfate (blue, n = 41) treated subjects. For each time period the consumption of paracetamol was lower in the reparagen treated group as well as cumulative consumption, depicted as week 0 – week 8 (*, p < 0.01). Results are depicted as the average number of tablets (500 mg) consumed per week.

The rescue medication was paracetamol (acetominophen). Consumption was relatively consistent throughout the study (Fig. 8), despite a reported decline in symptoms. However, significantly fewer paracetamol tablets were consumed in the reparagen group versus glucosamine at each assessment period, and for the total study (p < 0.01).

## Discussion

This study was designed to compare a new polyherbal therapy, reparagen, with glucosamine sulfate in mild to moderate osteoarthritis of the knee. Reparagen is derived from South American medicinal plants and supported by preclinical studies demonstrating chondroprotective, cytoprotective, and anti-inflammatory properties [21-24] as well as the ability to enhance human chondrocyte production of the cartilage repair factor, IGF-1 [20]. Additionally, one of the components of this polyherbal blend, vincaria (*Uncaria guianensis*) had successfully completed a small clinical study in osteoarthritis of the knee, with benefits within a week of administration at a three-fold lower doses than used in this trial [25].

Drop-outs from the study were at an acceptable rate, with the majority were due to unrelated illnesses or failure to be available for follow-up. There were no serious adverse events that were attributable to either reparagen or glucosamine, and tolerability was excellent. Subject randomization was effective, with entry and basal values equivalent in both groups and comparable to a previous study using the same experimental design and centers [46].

Results demonstrate that reparagen and glucosamine sulfate were able to elicit significant reductions in pain, stiffness and functional indices of osteoarthritis disease activity. These benefits were statistically significant within one week of treatment using the WOMAC and VAS criteria.

These observations using response rate as the primary efficacy variable (Figure 2) was supported by the secondary efficacy variables; reductions in baseline WOMAC scores, VAS pain, global assessments of outcome by both investigators and subjects were highly favorable, along with tolerance. Collectively, these results suggest that both reparagen and glucosamine sulfate provide effective relief of mild to moderate osteoarthritis of the knee in this population. The present study design did not include a placebo arm, and as such poses limits as to interpretation. However, in a previous nutraceutical trial that we performed in the same target population, using the same entry criteria and comparable sites and investigators, we noted response rates to placebo based on a 20% reduction in WOMAC pain were substantially less (4 weeks 32%, 8 weeks 59%) than what was observed in the present study using the same criteria [46].

Clinical trial results with glucosamine displays some variability, and the two recently performed large trials, GAIT and GUIDE, collectively frame this issue [11,12]. Given that in the present study we used glucosamine sulfate as a positive comparator without a placebo group, these issues have some bearing on the interpretation of the current study. In the GAIT study, results with glucosamine hydrochloride were indistinguishable from placebo at 4 and 24 weeks [11]. In contrast, the GUIDE protocol demonstrated that glucosamine sulfate was significantly more effective than placebo, whereas paracetamol was marginal in its benefits [12]. As glucosamine dose (1500 mg) was identical in both GAIT and GUIDE studies (and the present investigation) it is clear that total dose does not explain these variations. The present study does differ from both GUIDE and GAIT in terms of the timing and distribution of the doses. The GAIT study spread the daily dose out over three doses whereas the GUIDE used a once a day dosing regimen that is part of approved prescription approach in Europe. Because we were concerned that compliance would be compromised in this population with three times a day dosing and additionally as we wanted to match the dosing approach used for the ethnomedicines in reparagen we adopted the twice a day regimen. For glucosamine sulfate both three times a day and once a day regimens have clinical support for efficacy and safety [8,9,12,39-41] suggesting that timing is not a critical determinant of efficacy, but this present report does not include a pharmacokinetic analysis. We are not aware of direct comparisons where glucosamine sulfate efficacy has been evaluated with varied dosing regimens, but there is a report describing that chondroitin sulfate in a once a day regimen offers a similar efficacy as multiple doses a day [47], for the same total dose.

The magnitude of the glucosamine sulfate responses in the present study were greater than anticipated. Without an additional placebo arm it is difficult to frame these findings beyond the literature. One factor to be considered is ethnicity. The GUIDE study was European and the GAIT was performed in North America. Glucosamine sulfate, when administered in multiple daily doses, has previously shown to be effective in an Indian osteoarthritic population [39]. Thus, while ethnicity needs to be entertained as a factor in determining therapeutic potential, another consideration is the cultural influence of diet. Indian diets are characterized by the ubiquitous use of the spice turmeric, and with it the bioactive curcumin. There have been numerous studies that have defined an anti-inflammatory role for curcumin suggesting that it may offer benefits in the treatment of osteoarthritis [48-50]. These actions primarily center on the suppression of pro-inflammatory cytokines and catabolic factors like matrix metalloproteinases (MMPs) secondary to inhibition of gene expression via redox-dependent transcription factors [51,52]. Given that MMPs are responsible for the degradation of cartilage matrix and the release of glucosamine, suppression of MMP formation with dietary curcumin could enhance the effectiveness of supplemental glucosamine. We are not aware of any study that has directly assessed the combined actions of glucosamine and a transcriptional inhibitor that regulates MMP formation, so this conclusion remains unsupported. However, the combination of the antioxidant MSM, and glucosamine sulfate has been reported to be additive in Indian subjects [39]. Additionally, using markers of collagen type II breakdown, it has been proposed that subjects that have a high cartilage turnover may be more receptive to the benefits of glucosamine [53].

This potential influence of redox active factors present in the diet also reflects on the proposed mechanism of action for reparagen. Studies using explants of human cartilage indicate that catabolic degradation of cartilage matrix, a MMP dependent response, is suppressed by reparagen in concert with increased expression of the cartilage repair factor, IGF-1 [20]. High levels of glucosamine (mM) in explant studies have been shown to limit cartilage catabolism but in contrast to reparagen, anabolism was also compromised [37]. It is still unclear if this is the underlying basis for the benefits if glucosamine as in vivo administration of therapeutic doses only raises blood and joint levels to the low μM range [54-56], well below the concentration required to alter catabolic activity. Nevertheless, based on this proposed mechanism of action it would also be likely that the combination of reparagen with glucosamine sulfate may produce enhanced benefits. However, the focus of the present study, were the actions of these agents individually, not collectively.

IGF-1 is an important anabolic repair factor for joint health, promoting growth and repair of damaged cartilage [23-27]. While reparagen has been shown to promote IGF-1 production in cartilage explants, and negate the suppressive effects of IL-1β on IGF-1 production by chondrocytes [17], it is difficult to assess these events in a clinical trial. Serum levels of IGF-1 were assessed and were increased by 5% with either treatment, an effect that was not significant. Serum IGF-1 levels are thought to reflect hepatic production, and it is unknown as to what level changes in joint production would be reflected in circulating levels. From this study there does not appear to be a strong link between serum IGF-1 and joint health. However, it is worth noting however, that serum IGF-1 levels were low in this population when compared to large population studies in other settings [57-60]. Indeed, serum IGF-1 levels in this group were more reflective of subjects that were 20 years older. The reasons for this are unclear as the subjects were otherwise in good health and while rheumatic heart disease is associated with reduced serum IGF-1, rheumatoid arthritis is not [61]. It is also unknown if low serum IGF-1 levels enhances the effectiveness of reparagen or glucosamine, however, it does not appear to preclude their efficacy. Thus, we conclude that serum IGF-1 levels in absolute numbers or in terms of changes with intervention, were not predictive of outcome in these subjects.

A number of studies have compared glucosamine sulfate with non-steroidal anti-inflammatory drugs like ibuprofen as positive controls [37-39], without the inclusion of a placebo group. The advantage of this approach is that need for rescue medication is less and the data may not be as clouded by the mixed effects of both treatment and rescue medication. In these studies the outcomes generally reflect a favorable response to glucosamine sulfate in terms of safety and efficacy and have contributed to the growing interest in the nutraceutical management of arthritis. Nevertheless using glucosamine sulfate as a positive comparator is a controversial design given that the literature debates the effectiveness of different glucosamine formulations.

A consistent outcome was that reparagen treatment was associated with a reduction in the use of the rescue medication. (Fig. 8, p < 0.01). The lower consumption of paracetamol was significant at each assessment interval during the protocol. Given the concerns associated with excessive paracetamol consumption on liver function [48] or the deleterious effects of non-steroidal anti-inflammatory drugs (NSAIDs) on gastrointestinal, renal and cardiovascular function [62-65], the reduced paracetamol consumption with reparagen is worth noting. Additionally, the vincaria component of reparagen has been shown to block the severe gastrointestinal complications associated with high dose NSAIDs [21,23]. Thus, not only is this botanical component effective in treating arthritis it may also limit the side-effects of common pharmaceutical approaches to managing arthritis. In 28 day sub-acute safety and toxicity studies, reparagen displayed no evidence of toxicity and was given the top OECD safety rating of 5 or unclassifiable. The excellent tolerability and safety profile seen in this study confirms these OECD safety and toxicity studies and is consistent with the cultural history of safe use of the parent medicinal plants.

## Conclusion

Reparagen and glucosamine sulfate both produced a steady reduction in osteoarthritis symptoms, particularly pain, with continued improvements upon sustained treatment. Use of the rescue medication was significantly less in reparagen subjects, but otherwise responses were comparable between reparagen and glucosamine sulfate groups. Both investigational agents were well tolerated and safe. Reparagen should be considered an effective option in the management of osteoarthritis, particularly for those subjects with seafood allergy or diabetes.

## Abbreviations

MMP matrix metalloproteinase

IGF-1 insulin-like growth factor-1

NF-κB nuclear factor kappa B

AP-1 activating factor-1

NSAIDs non-steroidal anti-inflammatory drugs

GUIDE glucosamine Unum In Die Efficacy

GAIT glucosamine/chondroitin arthritis intervention trial

WOMAC western ontario and mcmaster universities osteoarthritis index

VAS visual analog score

OARSI osteoarthritis research society international

OMERACT outcome measures in rheumatology clinical trials

ARA american rheumatology association

## Competing interests

KM is an employee of Vedic Lifesciences, Pvt, Ltd., a CRO that performed the study.

ND is an employee of Vedic Lifesciences, Pvt, Ltd., a CRO that performed the study.

MJSM is an advisor to Rainforest Nutritionals, Inc who supported the study and for these services has been compensated with equity but no other financial compensation.

## Authors' contributions

KM contributed to study design and execution, manuscript preparation, data analysis and management

JG contributed to patient recruitment, evaluation, study execution and manuscript review

SB contributed to patient recruitment, evaluation, study execution and manuscript review

HT contributed to data analysis

MM contributed to patient recruitment, evaluation, study execution and manuscript review

SN contributed to patient recruitment, evaluation, study execution and manuscript review

ND contributed to study design and execution and GCP monitoring and protocol compliance.

MJSM reviewed data and contributed to manuscript preparation.

All authors read and approved the final manuscript.

## Pre-publication history

The pre-publication history for this paper can be accessed here:


